# Young stroke survivors’ experiences and approaches to community reintegration: a descriptive qualitative study

**DOI:** 10.1186/s12889-025-25918-9

**Published:** 2025-12-17

**Authors:** Suzanne Hoi Shan Lo, Janita Pak Chun Chau, Laveeza Butt, Marika Demers

**Affiliations:** 1 The Nethersole School of Nursing, Faculty of Medicine, The Chinese University of Hong Kong, New Territories, Hong Kong SAR; 2 School of Rehabilitation, Faculty of Medicine, University of Montreal, Montreal, Canada

**Keywords:** Community reintegration, Social participation, Qualitative research, Rehabilitation, Rehabilitation nursing, Stroke, Young stroke

## Abstract

**Background:**

Community reintegration is a key goal for younger survivors of stroke due to participation demands such as returning to work and caring for dependents. However, current post-stroke rehabilitation services largely cater to older survivors and lack targeted support to promote reengagement in life activities. This study aimed to explore the community participation experiences of young stroke survivors to better understand their recovery needs and provide insight into strategies to enhance community reintegration and post-stroke care services.

**Methods:**

A qualitative descriptive study was carried out. Twenty-one stroke survivors aged between 18 and 64 years were recruited from a community-based rehabilitation centre and stroke support group in Hong Kong. Individual semi-structured interviews were conducted to explore participants’ experiences of post-stroke community participation, associated facilitators and barriers, unmet health needs, and required support. Interview data were thematically analysed.

**Results:**

Participants were aged 55.5 (SD: 7.0) years on average, and had stroke for a mean of 8.1 years (SD: 6.5). Four main themes were identified: (1) self-expectations and personal drive to recover; (2) physical, psychosocial, and environmental obstacles; (3) personal strategies to overcome barriers; and (4) external facilitators and recovery needs. Overall, while survivors were motivated and expressed a keen desire to resume life engagement by taking up former responsibilities and activities, factors such as limited functional mobility, lack of age-appropriate rehabilitation services, and insufficient social and vocational support were notable barriers to participation.

**Conclusion:**

This study demonstrated that the resumption of community participation was a significant concern for younger stroke survivors, indicating that diversified stroke rehabilitation and support services are required to meet their age-based recovery demands. Return to work may be supported by timely vocational rehabilitation and promoting disability-friendly workplace policies, while volunteering opportunities could be provided as an alternative vocational activity. Furthermore, educational and psychosocial interventions to promote positive interpersonal relationships and strengthen survivors’ mental health would be helpful in facilitating participation outcomes.

**Supplementary Information:**

The online version contains supplementary material available at 10.1186/s12889-025-25918-9.

## Background

Stroke in younger adults accounts for over 15% of all stroke cases [1]. A recent systematic review and meta-analysis on global stroke prevalence suggests that adults aged under 55 years face a rising risk of stroke, with a Dutch study reporting a 23% increase in ischaemic stroke incidence among adults aged 18–44 years over a 10–year period [2, 3]. Disability arising from changes in survivors’ physical and cognitive abilities is a major long-term consequence of stroke, often impacting individuals’ functional independence, psychosocial health, and community reintegration for the rest of their lives. In particular, younger stroke survivors with lasting impairments may face greater challenges in adjusting to an altered life after stroke due to higher personal and societal expectations of participation and engagement with life roles in comparison with older survivors whose roles and activity demands may differ. Accordingly, between 30 and 80% of working age survivors report less satisfaction with their level of post-stroke participation compared with older survivors, reflecting younger survivors’ unmet needs regarding life resumption such as returning to employment, engaging in care of children or elders, and participating in leisure activities [4]. 

Indeed, qualitative investigations of younger survivors’ experiences reflect unaddressed anxieties about fulfilling various family and personal expectations while adjusting to post-stroke mobility or cognitive limitations [5]. Specifically, returning to work is a significant struggle as an increasing number of studies report that young survivors face difficulty in securing employment due to concerns such as insufficient disability accommodations, and potential discrimination by colleagues or employers [6, 7]. Consequently, associated fear and stress may not only lead to delayed or no work resumption, but also negatively impact survivors’ self-perceptions and self-value over time [8]. Similarly, shifts in personal roles due to losing the ability to care or provide for dependents such as children or elders are also reported to be a major cause of distress, frequently requiring survivors to renegotiate interpersonal relationships and even reconstruct their self-identity in attempts to adjust to their post-stroke reality [9, 10]. 

In Hong Kong, current stroke rehabilitation and support pathways are primarily geared to accommodate the needs of older survivors, focusing on functional recovery to improve basic self-care capacity and a degree of independent living. The core components of formal inpatient stroke rehabilitation thus usually include physiotherapy, occupational therapy, and speech therapy, in order to facilitate the hospital-to-home transition and promote independence in carrying out basic daily life activities. Moreover, vocational support services currently available are not specific to the needs of people with stroke and thus unable to adequately support their community reintegration. As a result, younger adults with varying rehabilitation needs report a lack of age-appropriate post-stroke care and support, reflecting a significant gap in stroke services [11]. Similarly, existing literature on experiences of participation and community reintegration after stroke also centres older survivors, thus necessitating studies with younger samples. As such, to identify younger survivors’ distinct recovery goals and develop relevant support services, this study explored the post-stroke recovery and participation experiences of community-dwelling stroke survivors aged under 65 years. Findings from this study are expected to inform the design and delivery of relevant community-based rehabilitation services for younger adults with the aim to promote positive recovery experiences and optimise their reintegration into society.

## Methods

### Aim

To explore the post-stroke recovery and community participation experiences of young stroke survivors (aged between 18 and 64 years) residing in the community.

### Design and setting

A qualitative descriptive study was conducted from May 2021 to April 2022 with participants recruited from a community-based rehabilitation centre and a stroke support group in Hong Kong. One-on-one semi-structured interviews were conducted in participants’ homes. This design was applied as it allows for deeper investigation of participant experiences and perceptions of the phenomenon under study [12]. The study findings are reported according to the Consolidated criteria for Reporting Qualitative Research guidelines [13]. 

### Participants

Participants were recruited through convenience sampling. The first author contacted and screened potential participants referred by the community centre and stroke support group through phone calls. Individuals were eligible if they: (1) were aged 18–64 years, (2) were diagnosed with a first-time or recurrent ischaemic and/or haemorrhagic stroke, (3) had a Montreal Cognitive Assessment score above the 2nd percentile [14], (4) lived at home, (5) were able to communicate in Cantonese. Individuals who had been diagnosed with a psychological disorder or had communication difficulties that severely limited their ability to converse with the researcher were excluded. Eligible individuals were invited to join the study and their written informed consent was obtained, followed by scheduling of the interview. No relationship with participants was established prior to study commencement.

### Data collection

A semi-structured interview guide was developed based on literature on community reintegration after stroke (see additional file “Interview guide”) [5, 11, 15–18]. Participants were asked to share their experiences of resuming community participation after stroke, including associated facilitators and barriers, any unmet health needs, and desired support. The first author piloted the interview guide with three eligible individuals and questions were well understood. The interview guide was not subsequently revised.

All interviews were conducted face-to-face in Cantonese by the first author and audio-recorded with consent. The first author is a registered nurse with a doctorate degree, over 15 years of experience in working with people with stroke, and extensive experience in conducting qualitative research [18, 19]. Interviews lasted for 45 to 110 min and three participants were accompanied by their spouse, children, or carer. Accompanying persons were requested not to comment on participants’ views nor express their own views to maintain participants’ independence in expressing their personal thoughts. During the interviews, the first author kept field notes with reflections on the responses and reactions of participants. Before concluding the interviews, the first author summarised the key points and confirmed their accuracy with each participant [20]. Any necessary changes were made and the data entries were finalised. Participants’ demographic and clinical information were then collected.

### Data analysis

Interview audio recordings were transcribed verbatim and anonymised. The first author confirmed the accuracy of the transcription by reviewing the interview transcripts and audio recordings. The six-step process of inductive thematic analysis was followed [21]. The first and second authors read all of the transcripts several times to familiarise themselves with the data. Independent coding of the data was conducted by highlighting specific portions of the transcribed data that addressed the research aim. The two authors then compared and discussed the generated codes after which they mutually agreed on the coding framework for the data collected through the transcripts. The framework was finalised after analysing six transcripts when no new codes could be identified. Preliminary themes were then identified from the codes and reviewed to ensure they accurately reflected the data. Finally, theme names were carefully considered and refined to ensure the best representation of the concepts being described.

### Rigor, trustworthiness, and data saturation

The strategies used to maintain the rigor and trustworthiness of the analysis included a member check (credibility), an audit trail (dependability), reflexivity (confirmability), and data saturation (transferability) [20]. Transcripts of each interview were presented to the respective participants for confirmation of completeness and accuracy in conveying their views. Research team members who analysed the data regularly revisited the audio recordings, field notes, and reflections to confirm the accuracy of the transcripts and identify any issues that may affect data analysis. All authors met regularly to discuss the coding system and review the appropriateness of the organisation of codes into determined subthemes and themes to ensure the best representation of the meanings and patterns. A transparent audit trail was maintained to document the decision-making process and author reflections that led to the development of the final themes. Data collection was concluded when the data from the last eight participants did not generate any new codes or themes, indicating that data saturation had been reached [22]. 

### Ethical considerations

The study received ethical approval from the Joint Chinese University of Hong Kong-New Territories East Cluster Clinical Research Ethics Committee (CREC Ref. No.: 2019.010). The principles of the Declaration of Helsinki were adhered to during the design and conduct of the study. All collected data were used for research purposes only and confidentiality was ensured by anonymising interview transcripts and using password-protected storage files.

## Results

Twenty-one individuals were approached to join the study and interviewed following screening for eligibility. Most participants were male (66.7%), married (71.4%), and first-time stroke survivors (95.2%), while one participant had multiple recurrent strokes. On average, age at the time of interview was 55.5 years (SD: 7.0), while age at the time of first stroke was 47.5 years (SD: 7.6) with a mean of 8.1 years (SD: 6.5) since stroke onset. The majority of participants had ischaemic stroke (61.9%). Though all participants were either employed or homemakers before stroke, only 23.8% had returned to employment post-stroke and over 85% required assistive aids. Table 1 shows the full demographic and clinical characteristics of the participants.

Four themes and ten sub-themes were identified (Table 2). The first author translated participant quotes from traditional Chinese to English as she is fluent in both languages. Each participant was referred to by an identifier from A1 to A21 and their age.

**Table 1:** Participant demographics and clinical characteristics (*N* = 21)

Participant Characteristics	*N* (%)
**Socio-demographic characteristics**	
Age at interview, years ^†^	55.5 (7.0), range: 36–66
Age at stroke, years ^†^	47.5 (7.6), range: 29–62
Time since stroke in years ^†^	8.1 (6.5), range: 1–22
**Sex**	
Male	14 (66.7)
Female	7 (33.3)
**Marital status**	
Married	15 (71.4)
Single	6 (28.6)
**Education level**	
Primary	1 (4.8)
Secondary	16 (76.2)
Tertiary	4 (19.0)
**Post-stroke employment**	
Employed	5 (23.8)
Homemaker	2 (9.5)
Retired	3 (14.3)
Unemployed	11 (52.4)
**Clinical characteristics**	
Type of stroke	
Ischaemic	13 (61.9)
Haemorrhagic	8 (38.1)
**Affected side of the body**	
Left	10 (47.6)
Right	11 (52.4)
**Assistive aids**	
Unaided	3 (14.3)
Stick	16 (76.2)
Wheelchair	2 (9.5)

Note: Data marked with ^†^ are presented as mean (standard deviation), range, all others are presented as frequency (%)

**Table 2:** Themes and sub-themes identified from interviews

Themes	Sub-themes
1. Self-expectations and personal drive to recover	1.1 Obligation to fulfil roles and responsibilities
	1.2 Seeking resumption of former independence and activities
	1.3 Gratitude for others’ care and support
2. Physical, psychosocial, and environmental obstacles	2.1 Fatigue and psychological barriers
	2.2 Poor community awareness and attitudes towards stroke
	2.3 Limited outdoor accessibility and discouraging experiences
3. Personal strategies to overcome barriers	3.1 Develop mental fortitude and persist with physical rehabilitation
	3.2 Take initiative to communicate and facilitate mutual understanding
4. External facilitators and recovery needs	4.1 Community and peer encouragement
	4.2 Age-appropriate and need-specific rehabilitation services

### Theme 1: Self-expectations and personal drive to recover

#### Obligation to fulfil roles and responsibilities

While participants noted stroke as a traumatic and disruptive experience which left them with feelings of uncertainty and despair in early stages of their recovery, they revealed several motivating factors which eventually helped them to overcome their negative thought patterns and strive to resume community participation. For instance, as several participants had young families with school-going children or parents who required care and assistance, they worried about becoming a burden on their loved ones, and being able to retake family responsibilities became a forefront concern for survivors.

*“Originally*,* I used to take care of my family*,* now [after stroke] it became my family having to take care of me… I wondered whether it was a burden on my family? I was really worried about this.”* (A5, Age 55).

*“…Every time I saw my family members and my wife- my daughter was still studying in secondary school… When I was genuinely considering giving up [on recovery]*,* a question would pop up in my mind- What would happen if I left them? My daughter loves me very much. She was relying on me. I worried about them and I felt sorry to my wife.”* (A16, Age 62).

Cultural and gendered expectations from participants were also highlighted as men without employment expressed regret at struggling to take on the role of the primary breadwinner while women felt similarly regarding their reduced contributions to managing their household.

*“This is my biggest hope and biggest motivation to continue living. I want to take up my responsibility as a husband again. Take care of my family and my wife. This is what I expect [of myself].”* (A4, Age 65).

*“…I felt like I owed my wife. Because I was unable to take care of her and she took care of me on her own instead.”* (A20, Age 36).

*“As a housewife*,* I could not do anything and even needed others to take care of me. I was unhappy…”* (A17, Age 59).

Returning to work was also a priority for most survivors as they felt too young to retire, with two survivors remarking that as they were in their 40–50 s, they did not expect to receive or rely on government assistance for the rest of their lives. Instead, most survivors considered working to be a way to support their families, become self-reliant, and an effective way to occupy and make use of their time.

*“If you are already 90 or 100 years old*,* no one will force you to exercise or go to work because it may feel harsh. But if you are just 40 or 50 years old… you expect yourself to still have some capacity. I have my arms and legs … to [support myself] in doing this task [work]. I don’t want to stop. The world keeps moving [forward] and only I’ve stopped there. I didn’t expect to be like this. I wanted to keep striving for my own path. Yes*,* [resuming work] definitely is hard*,* but it’s well worth it.”* (A7, Age 52).

*“I don’t want to be a burden to others*,* I want to do something. I want to do something constructive for my family. …I plan to go back to work after the coming lunar new year. The amount of work does not matter. … Better than staying at home… no salary when sitting at home.”* (A10, Age 52).

#### Seeking resumption of former independence and activities

Having experienced significant changes to their physical/cognitive health post-stroke, several participants lost their sense of independence and felt their original life plans were interrupted. One participant felt that his life had come to an abrupt pause as he had just gotten married and was doing well in his career prior to stroke. Considering their active social participation and perceived future life plans, participants deeply desired to resume their previous life activities where possible.

*“All of a sudden [after stroke]*,* I had to pause or cease all the things I could’ve done the plans I had made. I wish to be what I used to be and to do what I wanted to do. …I’m only in my thirties. There are still lots of possibilities in my life*,* and I’m able to do many things. There’s no reason for me to stop. So*,* I have to force myself to keep going.”* (A20, Age 36).

Participants also strongly believed in the necessity of being independent in self-care to avoid relying on others for support in their daily activities.

*“I think that … the most important thing is one must be able to take care of themselves with their own ability*,* because no one can take care of you forever. So*,* my biggest source of motivation was the belief that [I] have to be able to take care of myself.”* (A1, Age 55).

Notably, one participant recalled the experience of having nurses help her shower, which she felt stripped her of her dignity as she did not expect to require such basic help at a young age, noting that:

*“After the shower*,* I told myself that I will never let myself have this type of experience a second time.”* (A5, Age 55).

#### Gratitude for others’ care and support

Strong emotional and practical support received from family and close relatives reinforced participants’ resolve to actively reengage with their life as they felt thankful for their encouragement and did not want to disappoint their loved ones. Specifically, participants noted how family members willingly adapted to their situation and took on different responsibilities as a way to reduce their burden and allow them to focus on rehabilitation.

*“When my daughter and my younger brother visited me [in the hospital]*,* they told me not to think about anything and to only do the things that could help me recover. [They said] do not think about anything else*,* and that they would help me think about those issues. So*,* their support was the greatest.”* (A13, Age 51).

*“I think that as my wife supports me so much*,* I can’t let her down… she encouraged me a lot [in rehabilitation]*,* so I must work hard to recover. My wife didn’t give up on me*,* so I have no excuse to give up on myself. I have to work even harder to reach the goal of being able to care for myself so my wife doesn’t have to go through so much trouble.”* (A14, Age 57).

Consequently, several participants themselves also developed a social spirit and voiced out their wish to support peers or other needy groups in society, with volunteering being a common pursuit due to its rewarding nature and compatibility.

*“When I get well*,* I want to give back to society. If have the chance*,* I hope to do some volunteering activities… I want to find work that is light and suited to my abilities… provide more encouragement to other people with stroke and help them manage their situation after stroke.”* (A4, Age 65).

*“One day I received a phone call from a hospital [staff] asking me if I could go to the hospital and help the patients. I said yes. In that moment*,* my mood had been low [but] after I helped the patients*,* they made me better… when I witness their improvement*,* I feel happy.”* (A11, Age 60).

### Theme 2: Physical, psychosocial, and environmental Obstacles

#### Fatigue and psychological barriers

Despite participants’ resolve to resume their community engagement, a number of obstacles emerged, with limited post-stroke physical and executive function being a common barrier. Participants reported difficulty in walking long distances due to fatigue and fear of potential adverse events in public. A corresponding psychological impact was also revealed as survivors expressed lower confidence and self-consciousness regarding mobility.

*“… I also didn’t want others to see that my walking gait was a bit weird*,* it made me feel less confident.”* (A5, Age 55).

*“Since I might have a seizure… I won’t use the electric wheelchair. Because if I have a seizure*,* I will have problems easily… since I won’t be able to control it.”* (A14, Age 57).

*“I was quite worried when crossing roads. Sometimes my eyes couldn’t differentiate between the red and green traffic lights… I worried a lot during this time.”* (A3, Age 62).

The slow pace of recovery was also a common issue as a survivor noted how their plans to resume work had to be delayed as they found it challenging to keep up with tasks in fast-paced environments.

*“In terms of work*,* I originally thought that I would be able to resume in a short time… [but] there are not too many things that I am able to do. I still need more rest so I just put down the work for now… My left hand only [recovered] about 20 to 30%… I think the progress is very slow.”* (A9, Age 48).

#### Poor community awareness and attitudes towards stroke

Aside from managing physical limitations, participants were stressed by others’ limited understanding of stroke and the recovery process, leading to a sense of frustration. For example, as some family members did not understand differences in stroke severity and the uniqueness of individual health conditions, participants were expected to recover quickly due to their younger age, thus causing significant mental stress.

*“[My family] generally believed that [most people with stroke] could walk after one year. But the fact is it’s not like that. [Recovery] really needs a longer time. They also think that since you are young*,* your [recovery] won’t take much time…”* (A9, Age 48).

*“There may be some other people who had stroke that they [family] know about*,* [they would say] ‘that uncle could walk one week after the stroke*,* unlike you who still can’t walk.’ It is really stressful*,* but [you] can only take this as a form of care… as their love and concern.”* (A9, Age 48).

Participants also felt judged and looked down upon by colleagues, employers, or members of the public, noting discriminatory attitudes in various situations which made them feel unwelcome or isolated by society.

*“When I went out to look for a job… I took my walking stick with me to the interview. [The] boss looked at me like this [moves his head from up to down]. I felt very sad. I used to work under one person*,* and supervised many people. Now*,* I attend a job interview for a clerical role… and you treat me like this.”* (A7, Age 52).

*“I remember this one time I took the train*,* a man called me out for blocking his way*,* though I hadn’t done it on purpose. He kept on going and saying*,* ‘be careful*,* I will run you over.’…I felt miserable and sad.”* (A17, Age 59).

*“Indeed*,* it was a bit harsh for me when I learnt to drive again… I had to prepare for the driving exam… There was a time when this person stared at me*,* then I stared back at him as well. What was the problem? I wasn’t a dead person. It’s just that I had this condition [stroke]. I did not die.”* (A7, Age 52).

#### Limited outdoor accessibility and discouraging experiences

Going outdoors was also a challenge due to participants’ limited physical/cognitive function and the hazards of public transportation. Specifically, participants highlighted the significant mental load of having to plan their journeys prior to leaving home as they would have to look up routes, consider transportation options, check walking distances, and ensure opportunities/spaces for rest.

*“Taking the bus isn’t suitable for me. The steps are higher and I have to hold onto handrails [due to strong jerking movements].”* (A10, Age 52).

*“I have to step over the gap between the train and the platform…when I take the train to work…so I repeatedly practiced this movement at home using yoga blocks.”* (A5, Age 55).

Adverse incidents which compromised participants’ safety were reported, with a participant recounting the alarming experience of falling on a bus.

*“I used a walking stick to get into the bus… hoped that I’d find a seat to sit down but there was none*,* so I had to stand… held my walking stick using my left hand*,* and held the handrail with my right hand. But my hands didn’t have enough strength [to grip] so when the bus stopped suddenly*,* I just fell. I rolled to the end of the bus and lay on the floor. The other passengers were very worried about me*,* asking if I was okay… I acted calm*,* told others that I was okay… actually I was frightened.”* (A5, Age 55).

### Theme 3: personal strategies to overcome barriers

#### Develop mental fortitude and persist with physical rehabilitation

Participants paid attention to their thought patterns and recognised the need to regulate negative thinking which caused low mood and affected motivation. They articulated the necessity of developing an optimistic mindset in enabling them to move forward and take control of their lives.

*“You shouldn’t focus on the things you’ve lost*,* but on those you still possess… [which] may offer you hope and possibilities. By remembering this principle*,* I was able to forget the despair caused by the paralysis on the left side of my body. And I will do what I have to do.”* (A20, Age 36).

*“I think this disease is interesting because psychology is more important than [physical] function… If you have positive intentions*,* your recovery will be better and faster [and you won’t have] a bad temper because it’s futile… If you have a hard time*,* your caregiver will also have a hard time… So be steadfast*,* hard-working*,* [and] positive. There are so many issues to worry about*,* but some can’t be fixed….”* (A11, Age 60).

Participants explained that acceptance of their post-stroke condition was necessary to resume their life engagement. In particular, they looked forward to resuming previous hobbies or finding alternative activities in accordance with their new capacities, thus generating a sense of fulfilment.

*“…I enrolled to learn calligraphy. I just learned and learned for several years. I also sometimes do calligraphy at home. … As time passed*,* … many people think my writing is very good. So now I teach others as a tutor and practise with others*,* do it together…. seems like we can make some contributions.”* (A12, Age 54).

*“I used to be an energetic person*,* … now I have the will but not the energy… My hobbies have changed. In my spare time*,* I now prefer to listen to music*,* watch films*,* and do some sedentary activities. I recently learned how to use computers… learned myself online … and study on the Internet…. Because [security] control rooms demand computer skills… So*,* if I comprehend this information*,* I may one day be in charge of the control position or aid in inspecting the control room.”* (A8, Age 51).

Being persistent with physical rehabilitation was also emphasised to facilitate functional recovery.

*“I go to the basketball court every night. There are boundaries on all sides of the court [which] I follow for four or five laps. I still [try to] walk quickly. As balance is impaired following a stroke*,* you might trip or fall if you move quickly*,* but you must try everything… otherwise you’ll be like this for the rest of your life… Although I still walk sluggishly*,* I am considerably better than before and no longer use a walking stick.”* (A8, Age 51).

*“I did physiotherapy every day*,* but I found myself in pain and exhausted afterwards. You must persevere and continue with the second*,* third and fourth round of physiotherapy… Actually*,* every time you stop [the treatment]*,* recovery will be pushed back one step [and] it seems like you have to start all over again. So*,* I had to be determined*,* never stop…”* (A5, Age 55).

#### Take initiative to communicate needs and facilitate mutual understanding

As low public awareness of stroke survivors’ needs could result in potential misunderstandings or interpersonal tensions, participants remarked that clear communication was key to helping others develop a better understanding of their condition and maintain cordial relationships.

*“When I sat down in the office and worked*,* [someone] looked at me… minded a lot about what I was doing. I asked him at lunch time one afternoon*,* ‘why are you always looking at me?’. It [turns out] that he was worried about me … if there was anything that I wasn’t able to handle*,* [he] wanted to help me. So*,* I told him frankly*,* ‘if there’s anything [that I need help with]*,* I will ask for your help*,* please don’t worry’. … Definitely I can’t move things… But when working*,* using pens*,* taking calls*,* other things*,* basically I can do those. After explaining that to the colleague*,* he no longer had other special things to ask me and didn’t look at me differently. I also felt very happy [that] we resolved this harmoniously.”* (A7, Age 52).

*“[Others] may not be discriminating … they just don’t know how to approach your [stroke] … some may worry that they might hurt you by saying something … I have to take the first step … join their social circle … take the initiative to have lunch with my colleagues … communicate with them … they just treat you like everyone else … they actually don’t mind waiting for me as I walk more slowly.”* (A1, Age 55).

Participants would also signal their need for accommodation in public spaces through gesturing to drivers to wait at road crossings and using assistive aids to raise the alertness of surrounding pedestrians, particularly for those with invisible disabilities.

*“I suggest that…when going outdoors… bring a stick. It gave me a sense of security when walking*,* allowing others to know that you have difficulties in walking…. in crowded places…By holding a stick*,* others can … be alert to [your condition]*,* and avoid bumping into you. It’s important because you need to be able to protect yourself…”* (A1, Age 55).

### Theme 4: external facilitators and recovery needs

#### Community and peer encouragement

Besides personal strategies, participants emphasised the importance of social support in facilitating their community reintegration, noting the positive impact of encouragement from family, friends, and peers, on their mental health and motivation for recovery. For instance, one participant recalled a memorable experience of appreciation from fellow marathon runners:

*“I was at the very end of all the runners [in a Marathon] and there was a staff member behind me urging us to speed up… But the runners on the opposite [side] all saw me and applauded me as encouragement. The sun was shining from there*,* and I felt so handsome in the sunlight.”* (A20, Age 36).

In particular, the value of peers as role models was identified as beneficial to their recovery since they could seek their help to improve their rehabilitation and provide mutual assistance in their recovery journeys, allowing them to access both psychological and practical support.

*“When I was training at the hospital*,* I got to know some peers*,* some of whom had a more severe stroke than me. [One] told me about some non-governmental organisations in the community that could help me with rehabilitation. He invited me to join a peer WhatsApp group … [which has] a lot of information about stroke.”* (A4, Age 65).

Survivors also noted the benefits of being able to connect with peers in similar age groups.

*“In the rehabilitation centre*,* I made some friends*,* all had stroke and we could understand each other well. [That] I can speak like this now*,* I must thank them because they helped me to speak more. Since they were young*,* some in their 30s*,* 40 s and 50s*,* we chatted freely… we talked about anything*,* sometimes about eating*,* food … current issues… They joked with me that I had improved greatly as I could speak foul language clearly. … Although I met my friends less after stroke*,* I’d meet more with these peers… provide positive reinforcement.”* (A2, Age 48).

#### Age-appropriate and need-specific rehabilitation services

A mismatch between existing rehabilitation services and survivors’ needs was reported. One participant recalled the disconcerting experience of having to stay in an old age care facility to recover despite their young age, pointing out a lack of age-appropriate rehabilitation options.

“*It was a strange sight for [the residents] in the old age home that I was staying there although I was still young. But the fact was that I had no choice. To keep my family reassured*,* I chose to go to the old age home to receive care. Psychologically*,* I couldn’t get used [to staying there] at the beginning and felt a bit uncomfortable.”* (A7, Age 52).

Given that resuming work was a challenge for most participants, assistance in preparing to return to the job market and searching for appropriate employment opportunities was required. Vocational counselling services were particularly appreciated as participants could explore alternative careers by discussing their specific needs or required accommodations, and find suitable work matched to their abilities.

*“Indeed*,* I was a bit lost at that time [of stroke] … It is lucky that the centre also offered vocational counselling in addition to other rehabilitation services. [The staff] also asked me about what kind of jobs that I wanted to do in future….”* (A7, Age 52).

*“I went to the [rehabilitation] centre and … a social worker discussed with me about what I could do [for work]. I chose to do customer service… I do not have to go out*,* no need to move things.”* (A7, Age 52).

*“I also hoped that I could find a job*,* but more like on a part-time basis*,* not full-time. Also*,* the location must be around the neighbourhood where I live. I had some peers with stroke*,* who had… rehabilitated quite well who told me about some training [jobs]*,* which were part-time and short term*,* offered in the community rehabilitation network for people like me who had a stroke… If I have the opportunity in future*,* I will try to ask for these jobs.”* (A2, Age 48).

## Discussion

Improving age-appropriate rehabilitation services and post-discharge pathways for young stroke survivors is a key development priority for the public health system in Hong Kong [23]. In this study, we explored the ways in which young stroke survivors navigated the resumption of community participation, with findings highlighting their mental resolve to adapt to post-stroke challenges and reintegrate in the community through resuming valued life activities, as well as notable barriers to participation such as gaps in rehabilitation services and poor community awareness of stroke recovery.

As participants had a high degree of independence and life involvement prior to stroke, they expressed a strong desire to be self-sufficient and reassume previous responsibilities in order to fulfil self-expectations as well as those of their families. This sentiment is commonly reported in previous literature on young stroke survivors’ motivations for recovery, which include conducting self-care, caring for dependents, and returning to work, all of which are also associated with reconstruction of post-stroke identity [15, 24]. Particularly since the perceived disruptive effect of stroke may be greater when it occurs at an earlier stage of life, younger survivors often may be more concerned about their recovery prospects and level of post-stroke participation compared to older survivors [4]. There is thus a need to recognise age-based variations in post-stroke priorities and recovery goals, and provide streamlined rehabilitation services in light of a growing population of younger survivors seeking to resume former levels of life participation.

Assistance in returning to work was identified as a core necessity as less than 25% of the sample had resumed work, which is notably lower than figures in studies from other regions (72% and 59% one-year post-stroke) [25, 26] especially considering that participants’ average time since stroke was 8 + years. Functional disabilities such as chronic fatigue, and social factors such as workplace discrimination were reported as major barriers, signalling the necessity to foster disability-inclusive attitudes and provide favourable workplace conditions such as flexible working hours and environmental modifications to accommodate survivors’ needs [27]. The merits of vocational rehabilitation were also noted as some participants reported that career counselling services helped them explore alternative career paths and find jobs compatible with their post-stroke abilities. However, further research on the optimal format of vocational rehabilitation is warranted due to heterogenous intervention approaches ranging from focusing on functional recovery and skill building to providing direct support in liaising with potential workplaces [28, 29]. Moreover, despite the introduction of government-subsidised schemes to support the hiring, training, and employment of people with disabilities, a recent study indicates that their effect is limited, with service users being confined to an environment of “segregated inclusion” in social enterprises and unable to secure employment in the open market [30]. This reflects a significant gap in vocational services, particularly for younger survivors who may expect and wish for a return to work similar to their pre-stroke jobs. Early identification of survivors’ career goals and expectations should therefore be encouraged during rehabilitation so as to design recovery plans that would enable them to find suitable employment opportunities. In addition, more advocacy work to promote inclusive workplace policies within wider society is required to further improve young survivors’ employment outcomes and overall workplace integration.

Besides formal employment, survivors expressed interest in volunteering as a type of vocational engagement, finding the activity to be emotionally fulfilling and more compatible with their post-stroke abilities. In line with international guidelines on supporting community reintegration [17], it would therefore be worthwhile to evaluate survivors’ diverse vocational interests and goals as part of their rehabilitation plan, and assist them through career counselling or liaising with relevant organisations to match them with suitable volunteering/work opportunities.

The impact of psychological health on resumption of life activities was also emphasised in this study. For example, though survivors recognised the need to adopt a positive mindset to move forward from stroke, struggles with self-consciousness about disabilities, low confidence in self-management, and fear of adverse events in public settings persisted and limited their social participation. Psychological management strategies should therefore be provided in tandem with conventional physical rehabilitation in order to mentally prepare and equip survivors to tackle common participation challenges. In particular, self-efficacy should be regularly evaluated and promoted after stroke given its mediatory role in the association between survivors’ mobility and level of social participation [31]. There is also promise in psychosocial interventions as findings from a Norwegian study suggest that guiding survivors to develop positive perspectives through regular empathetic dialogue with trained healthcare professionals encouraged post-stroke adjustment and life engagement [32]. Considering that the aim of post-stroke rehabilitation in Hong Kong mainly revolves around survivors’ physiological recovery, it is worthwhile to develop and integrate relevant psychosocial interventions to address the mental health needs of younger survivors and limit the negative effects of potential mood disorders on their community reintegration.

Social support has previously been found to be a strong predictor of post-stroke reintegration and was also highlighted by participants [33, 34]. Specifically, receiving emotional or practical support from family allowed survivors to feel reassured, accepted, and more motivated to reengage in life activities. On the flip side, survivors recounted frustration arising from others’ poor understanding of stroke recovery, which is also consistent with emerging evidence and underscores the necessity to strengthen health education on stroke within the wider community [35]. Particularly with regards to survivors’ families or caregivers, relevant age-specific educational support services should be provided to nurture mutual understanding, alleviate potential interpersonal conflicts, and reinforce survivors’ support network. A recent systematic review and meta-analyses reported that dyadic interventions for stroke survivors and caregivers could benefit outcomes of physical function and quality of life [36], indicating that caregiver or family-targeted interventions could promote survivors’ recovery through fortifying interpersonal relationships and long-term support systems. Furthermore, besides interventions targeted at survivors and their personal support network, it is also vital to promote inclusive values and behaviours in the society on a larger scale in order to reduce public discrimination and encourage the development of empathetic and supportive attitudes.

Survivors’ physical limitations and low accessibility in public spaces also limited their capacity for community participation. As most participants had mobility restrictions and relied on assistive aids, taking public transportation such as the bus or metro was a significant challenge as they struggled with balance, grip, and gait, indicating a need to formally assess survivors’ ability to use public transportation and develop targeted rehabilitation plans [37]. The importance of staying persistent with physical training to make progress in mobility recovery was further noted by survivors and reflected in the literature [38], indicating that adherence to regular physical activity should be encouraged post-discharge to promote mobility-related functional outcomes and support survivors in resuming outdoor activities.

A need for age-appropriate care facilities and strategies was also reported, reflecting unmet service demands and a sense of alienation among younger survivors [39]. According to the Hospital Authority, which manages all public hospitals in Hong Kong, younger stroke survivors had longer lengths of hospital stays (60.9 days) on average as compared with older survivors (43.7 days) due to the absence of day rehabilitation services that could meet their specific service needs upon discharge [40]. This is reflected in our study as participants voiced a need for diversified care and rehabilitation options, favouring strategies with social aspects such as peer support groups where they could connect and exchange valuable health information. Indeed, peer survivors were considered to be a valuable source of post-stroke assistance, with several participants noting that friendships developed with fellow survivors in early stages of rehabilitation were strongly conducive to their mental health as well as functional recovery, adding to growing evidence on the effectiveness of peer support in improving survivors’ overall health and rehabilitation outcomes through role-modelling and relevant knowledge exchange [41–43]. As such, investigating the recovery experiences and participation demands of younger stroke survivors provides directions for the planning, development, and integration of appropriate post-stroke rehabilitation strategies to better support their long-term journeys of community reintegration.

### Limitations

This study has some limitations. First, as participants were recruited by convenience sampling in a community-based rehabilitation centre and a stroke support group, the findings may not be applicable to young stroke survivors in other settings where services may differ. Second, although the term “Young stroke” usually describes stroke incidence in adults aged 55 years or below, our study widened the definition to also include adults up to the age of 64 years due to the ageing demographics of Hong Kong, which have meant that older adults make up a significant portion of the workforce and also require support with returning to work post-stroke. Third, stroke survivors with severe restrictions in communicating were excluded. Fourth, findings may be affected by recall bias since several years had passed since stroke onset for several participants. Finally, the study was based solely on the views of stroke survivors and perspectives of other individuals who may have played vital roles in their recovery, such as family, friends, or colleagues, were not explored.

### Implications for practice and research

Given that young stroke survivors have to manage impairments for longer periods of their lives, rehabilitation programmes should be long-term and extend beyond the acute phases of recovery in order to provide continuous support once survivors return to community settings. Moreover, individual participation priorities and goals should be identified and relevant assessments of their capacity to resume participation, such as mobility and confidence in taking public transportation, should be conducted at an early stage to inform individual rehabilitation plans. Psychosocial care should also be prioritised to strengthen survivor resilience and assist them in overcoming mental barriers to participation challenges. Furthermore, educational interventions aimed at key stakeholders such as caregivers and employers should be provided to promote social inclusion and foster empathetic attitudes and policies towards people with disabilities. Future studies may explore the community reintegration experiences of young stroke survivors with other functional disabilities such as aphasia. To further enrich the data, studies may consider using a combination of interviews and non-participant observation of how survivors manage participation challenges in real time. Quantitative analyses of changes in participation outcomes using validated instruments may also be helpful.

## Conclusions

In conclusion, this study found that younger stroke survivors were eager to resume life participation through responsibilities such as caring for their families and finding work. However, notable challenges included mobility restrictions, poor community awareness of stroke, and insufficient age- and needs-appropriate rehabilitation services. As such, survivors’ recovery plans should incorporate timely physical, psychological, and vocational assessments in order to formulate efficient rehabilitation plans aimed at achieving their ideal level of post-stroke participation in life activities. At the same time, disability-inclusive attitudes, facilities, and policies should be fostered within the wider society to increase social support in various contexts and promote optimal community reintegration experiences.

## Supplementary Information

Supplementary Material 1.

## Data Availability

The data that support the findings of this study are available from the corresponding author upon reasonable request.
