# Supplementary material for: Young stroke survivors’ experiences and approaches to community reintegration: a descriptive qualitative study
Source: BMC Public Health. 2025 Dec 17;26:269. doi: 10.1186/s12889-025-25918-9 (PMC12821175; doi:10.1186/s12889-025-25918-9)
Supplement: Supplementary file 1 — Supplementary Material 1. [file 12889_2025_25918_MOESM1_ESM.docx]

**Semi-structured interview guide**

1. Tell me your experience of returning to your pre-stroke life and community activities after being discharged home after stroke.
2. Tell me your experience of physical health needs when preparing yourself to participate in daily community activities, and former roles and responsibilities.
3. Tell me your experience of social health needs when preparing yourself to participate in daily community activities, and former roles and responsibilities.
4. Tell me your experience of psychological health needs when preparing yourself to participate in daily community activities, and former roles and responsibilities.
5. What health needs were met?
6. What health needs were unmet?
7. In your point of view, how could we address unmet health needs?
8. From your experience, what are the challenges in addressing unmet health needs?
9. In your point of view, what opportunities/facilities/strategies are required to encourage community reintegration among community-dwelling stroke survivors?
10. Do you have any additional concern(s) which we haven’t mentioned that you would like to share?
